# New insights into raceway cultivation of *Euglena gracilis* under long-term semi-continuous nitrogen starvation

**DOI:** 10.1038/s41598-023-34164-1

**Published:** 2023-05-02

**Authors:** Ranjith Kumar Bakku, Yoshimasa Yamamoto, Yu Inaba, Taro Hiranuma, Enrico Gianino, Lawi Amarianto, Waleed Mahrous, Hideyuki Suzuki, Kengo Suzuki

**Affiliations:** 1 Algae Energy Technology Research Institute, 649-17 Nishiyama, Taki-cho, Taki-gun, Mie 519-2171 Japan; 2 Euglena Co., Ltd., G-BASE Tamachi 2nd and 3rd Floor, 5-29-11, Shiba, Minato-ku, Tokyo, 108-0014 Japan; 3 Microalgae Production Control Technology Laboratory, RIKEN 1-7-22, Suehiro, Tsurumi, Yokohama, Kanagawa 230-0045 Japan

**Keywords:** Biofuels, Photosynthesis, Biotechnology

## Abstract

This study aimed to investigate the physiological responses of *Euglena gracilis* (*E. gracilis*) when subjected to semicontinuous N-starvation (N−) for an extended period in open ponds. The results indicated that the growth rates of *E. gracilis* under the N− condition (11 ± 3.3 g m^−2^ d^−1^) were higher by 23% compared to the N-sufficient (N+, 8.9 ± 2.8 g m^−2^ d^−1^) condition. Furthermore, the paramylon content of *E.gracilis* was above 40% (w/w) of dry biomass in N− condition compared to N+ (7%) condition. Interestingly, *E. gracilis* exhibited similar cell numbers regardless of nitrogen concentrations after a certain time point. Additionally, it demonstrated relatively smaller cell size over time, and unaffected photosynthetic apparatus under N− condition. These findings suggest that there is a tradeoff between cell growth and photosynthesis in *E. gracilis*, as it adapts to semi-continuous N− conditions without a decrease in its growth rate and paramylon productivity. Notably, to the author’s knowledge, this is the only study reporting high biomass and product accumulation by a wild-type *E. gracilis* strain under N− conditions. This newly identified long-term adaptation ability of *E. gracilis* may offer a promising direction for the algal industry to achieve high productivity without relying on genetically modified organisms.

## Introduction

*Euglena gracilis* is a unicellular freshwater motile alga belonging to the protist family Since its discovery in 1660s^1^, it has received considerable attention. *E.gracilis* is an important model organism for understanding photosynthetic mechanisms and eukaryotic cellular processes, owing to its unique properties of plastid and chloroplast organization^2,3^. Under aerobic-light conditions, *E. gracilis* undergoes photosynthesis and stores its energy in the form of a storage polysaccharide, unbranched β-1-3-glucan known as paramylon^3,4^. In anaerobic dark conditions, *E. gracilis* converts the paramylon into wax esters. These are single-chain lipids composed of saturated fatty acids (C14:0 myristic acid, C16:0 palmitic acid, and C18:0 stearic acid) and alcohols (myristyl alcohol)^4^. The applications of *E. gracilis* and its bioproducts (paramylon, and wax esters) are found in various fields such as dietary fiber, diabetic treatment, improving gut microbiota, food supplements, and biofuels^5–7^. Given its wide-ranging applications, *E. gracilis* has been established as a promising industrial microalga. Several algae-based industries use it for large-scale production of food, healthcare products, and biofuels^3^.

The algal industry faces a persistent challenge of achieving high biomass and bioproduct productivity with low operating costs. While there are potential benefits of algae in fields such as biofuel production, wastewater treatment, carbon capture, and climate change mitigation, their productivity remains a long-standing barrier to the industry^8–10^. Although, modulation of environmental conditions and nutrients can enhance bioproduct formation, it often results in decreased biomass productivity^7^. In the case of *E. gracilis* paramylon is considered a valuable bioproduct. Its accumulation has been observed under various conditions such as nutrient starvation, high salinity, electrical stimulation, co-culturing with bacteria, and heterotrophic cultivation^5,11–16^. Researchers have also attempted genetic modification to improve productivity and paramylon content in *E. gracilis*^17,18^. Despite such efforts to enhance biomass and bioproduct production, there is always a high price to pay in terms of technology or environmental impact.

Nitrogen starvation or limitation (N−) is a cost-effective and safe treatment to induce bioproduct accumulation in *E. gracilis*^19^. The treatment triggers metabolic changes that enhance the recirculation of (photosynthetically fixed) carbon from proteins to storage components like lipids or starch, resulting in an energy storage mechanism^20–22^. However, biomass productivity is typically reduced under N− condition compared to control conditions^22–24^. Various nitrogen treatment studies, such as intermediate addition, two-stage, semi-continuous, and sequential nitrogen starvation have been conducted to improve both biomass and lipid productivity^25–29^. Nonetheless, this theme requires to be elucidated further. A recent study on chlorella using a two-stage nitrogen starvation in batch cultures proved to be an efficient way to maintain lipid-rich high biomass^29^. Only a few studies have been conducted on *E. gracilis*, which revealed that the organisms biomass productivity decreases under N-limited conditions^29–32^. Only a few studies on genetically modified strains succeeded in achieving both high biomass and bioproduct accumulation under N-limitation conditions^17,33^. However, more research is necessary to improve the efficiency of the nitrogen treatment strategy to achieve high biomass and bioproduct productivity simultaneously.

**Figure 1:**
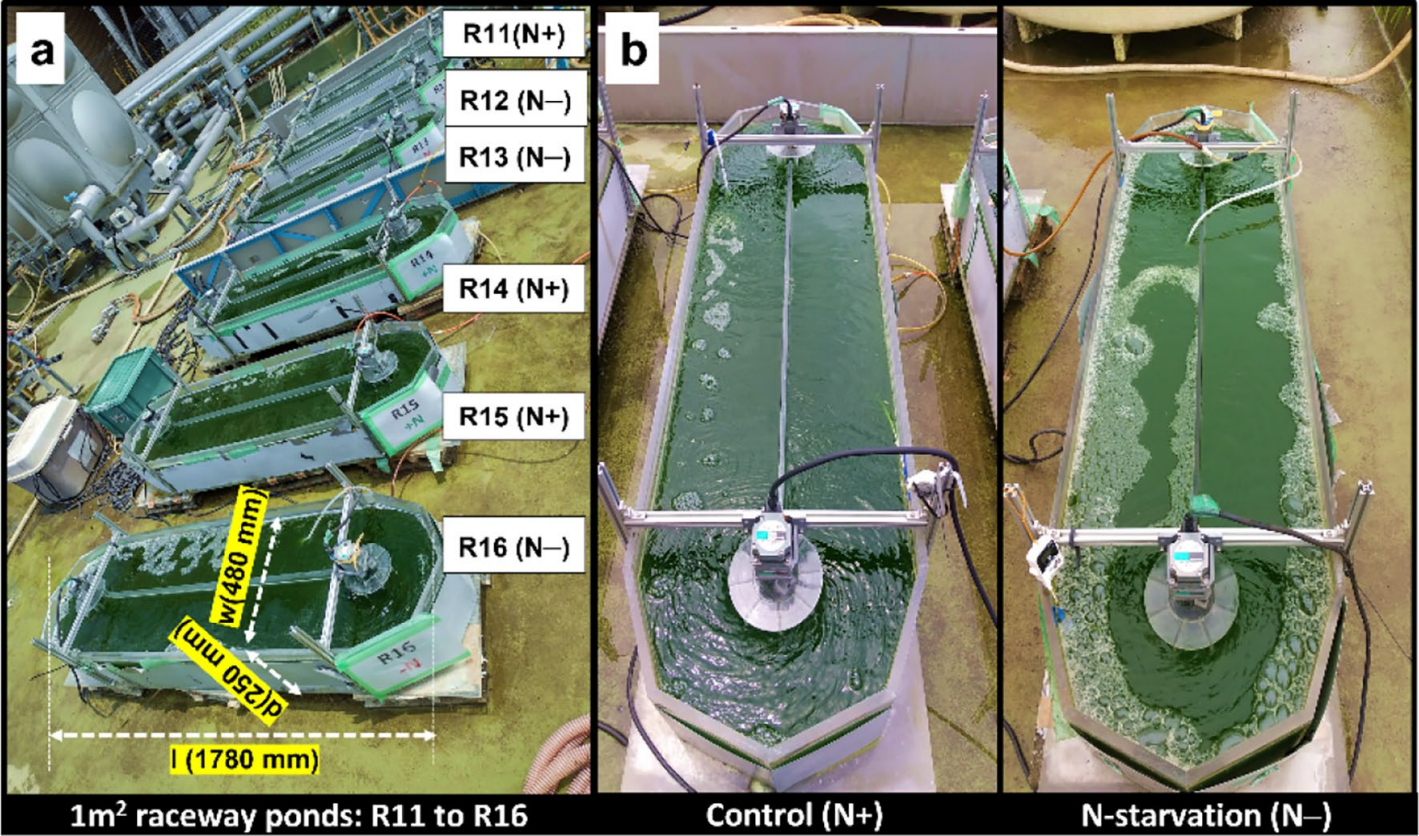
1 m^2^ cultivation system used in this study. (**a**) Raceway pond setup showing ponds R11 to R16. R11, R14, and R15 were maintained in control (N-sufficient, N +) and R12, R13, and R16 maintained in N-starved (N−) conditions. Here, l, d, and w, of the ruler represent the length, width, and height of the pond (in millimeters, mm) within the pond wall. The image was taken on day-1 of cultivation. (**b**) A comparative view of N+ and N− cultures in the raceway ponds. The images were taken on day-5 of the experiment.

Currently, very little is known about how *E. gracilis* responds to semi-continuous N− conditions, especially over long durations. Moreover, there have been no reports of such studies conducted in natural environments using *E. gracilis*. Given the importance of *E. gracilis* in industry, it is crucial to achieving stable semi-continuous cultivation and high productivity^34^. In light of this, our study aimed to investigate the growth and accumulation of the bioproduct paramylon in *E. gracilis* under semi-continuous N− conditions using open raceways (Fig. 1a,b). The findings of this study could be valuable in evaluating the feasibility of using semi-continuous N-cultivation as a method for cultivating *E. gracilis* for commercial production on a large scale.

## Results

### Semicontinuous system, nitrogen availability, and environmental changes

**Figure 2:**
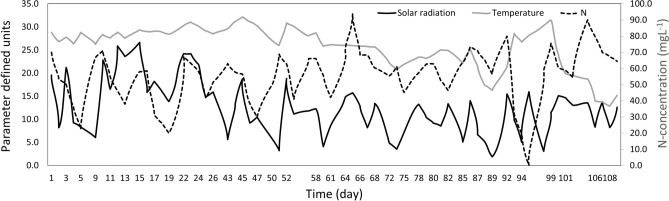
Mean N-content from N-sufficient (N +) condition, daily temperature, and solar radiation. The black, grey, and dashed lines represent solar radiation (MJ m^−2^ day^−1^), daily average temperature (°C), and N-content (mg L^−1^) from N+ ponds respectively.

Cultures were successfully maintained for 16 weeks without the addition of new seed culture during the four-month cultivation period. Experiments were conducted on a 5-day weekly basis for the N− condition with nitrogen availability for two days, followed by N-starvation for three days. Under the N+ condition, up to 20–60% of the initial nitrogen was consumed by day 5 (Fig. 2, Supplementary Fig. 1A). In N− ponds (R12, R13, and R16), the nitrogen was completely consumed by day 3, and the cultures were maintained under N− until day 5 (Supplementary Fig. 1A). It appears, that the amount of N consumption varies due to other environmental parameters as well. The temperature and solar radiation varied with time as the experiment progressed towards the end of 16 weeks. A decrease in average solar radiation and temperature was observed during rainy days and the beginning of winter (Fig. 2). This was reflected in nitrogen consumption correlation data as well (Supplementary Fig. 1B). N-utilization showed a moderate positive correlation between solar radiation (r = 0.3) and temperature (r = 0.4). Additionally, a moderate positive correlation between solar radiation and temperature was also observed, while they were negatively correlated with time.

### Growth

**Figure 3:**
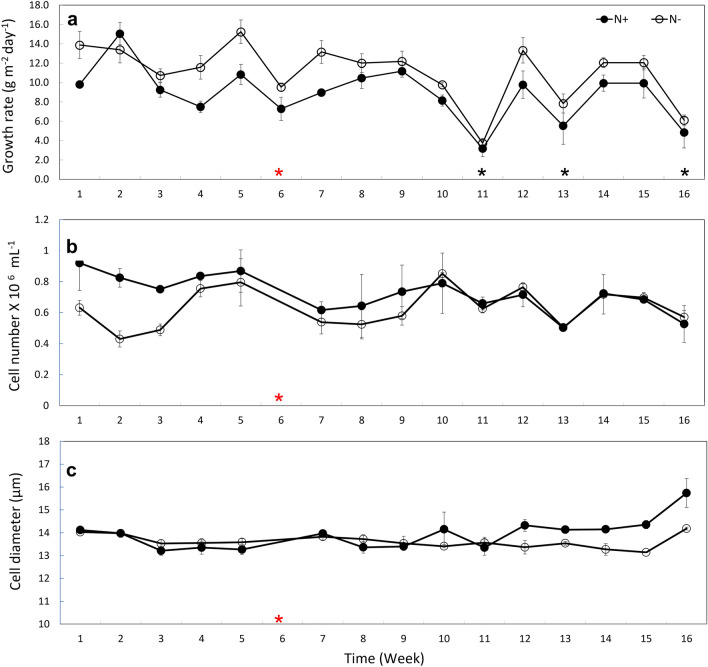
Growth Characteristics of *E. gracilis* under N-sufficient (N +) and, N-starvation (N−). (**a**) Comparison of weekly average growth rates between N+ , and N− . (**b**) Cell number, and (**c**) cell diameter at the end of each week of the weekly semi-continuous N− experiment. Empty circles indicate cells grown under N− condition and filled circles indicate cells grown under the N+ condition. Black asterisks indicate times of low growth rate. Red asterisks indicate weeks when cell number, diameter, and paramylon were not measured. Error bars for each measurement indicate the standard deviation of the mean values for each treatment (N+ and N−) across three ponds (n = 3).

The growth rate in the N− condition was higher (23%) than in the N+ condition (Fig. 3a). Weekly average growth rates were between 10–14 g m^−2^ d^−1^ in the N− condition, and 6–12 g m^−2^ d^−1^ in the N+  condition. The average growth rate for the weekly cultivation throughout the experiment in N+  and N− conditions were 8.9 ± 2.8 g m^−2^ d^−1^, and 11 ± 3.3 g m^−2^ d^−1^, respectively. From the statistical analysis, the growth rate data from day 1 indicate no significant difference, and the *p*-value tends to decrease over time from day-1 (G-DF: growth data frame, G-DF1) towards day-4 (G-DF2) (Table 1, Supplementary Fig. 2A). A significant difference in growth rates can be observed by day-5 (G-DF3) between the two conditions (Table 1, Supplementary Fig. 2B). There were clear differences in the growth performance between the conditions (Supplementary Fig. 3A, B). Over the course of 4 months, the cell count in the N+  condition was initially higher than that of the N− condition, but the difference gradually decreased over time. By week 10, the cell count in both conditions appeared to be similar (Fig. 3b, Supplementary Fig. 4A).

**Table 1:** One-way ANOVA analysis results of growth rate and cell diameter under N-sufficient (N** +**), and N-starved (N−). G-DF and D-DF represent data frame (DF) composed of growth rate (G) and cell diameter (D), respectively. DF1, DF2, and DF3, represent data on day 1, days 2–4, and all 5 days, of the 16-week experiments.

Data type	Data frame	Mean ± standard deviation		One-way ANOVA (*p*-value)
		N+	N−	
Growth rate (g m^−2^ day^−1^)	G-DF1	9.1 ± 7.8	10.5 ± 6.7	0.6
	G-DF2	8.9 ± 7.3	11.4 ± 7.0	0.1
	G-DF3	9.0 ± 7.4	11.1 ± 6.9	0.0*
Cell diameter (µm)	D-DF1	14.4 ± 0.7	14.2 ± 0.7	0.2
	D-DF2	14.0 ± 0.7	13.6 ± 0.4	8.2 × 10^−9^***
	D-DF3	14.1 ± 0.7	13.8 ± 0.6	1.0 × 10^−7^***

Significance levels are denoted by asterisks accompanying the *p*-value, with *** representing *p* < 0.001, ** representing *p* < 0.01, and * representing *p* < 0.05.

### Cell diameter

The cell diameter on the other hand was significantly different in diameter data frame D-DF2 and D-DF3 (Table 1). The cell diameter in the N− condition was similar to N+  in the first few weeks, and then it appeared to be smaller than N+  cells (Fig. 3c, Supplementary Fig. 4B). The pattern of changes in cell diameter appeared to vary from day 3 to 5 of each weekly experiment (Supplementary Fig. 4B). The diameter of the cells grown under both conditions (N+  and N–) was positively correlated with each other (r = 0.7) and with time (r = 0.3 and r = 0.0, respectively) as it progressed week by week in the 14-week experiment (Supplementary Fig. 4C). Furthermore, the cell diameter of N+  and N− conditions are negatively correlated with temperature (r =  − 0.4 and r =  − 0.1, respectively) and time as it progressed day by day within a weekly experiment (r =  − 0.2 and r =  − 0.1, respectively) (Supplementary Fig. 4C).

Overall results indicate that the cell diameter under N− conditions reduced slightly than the initial weeks, and was comparatively smaller than the N+  cell diameter. Nevertheless, the average cell number (0.6 ± 0.1 × 10^6^ for N+ , 0.6 ± 0.0 × 10^6^ cells mL^−1^ for N–) and diameter (14.2 ± 0.7 µm for N+, 13.8 ± 0.6 µm for N–) throughout the experiment were higher in the N+ condition than in N− condition (Supplementary Fig. 4D). A similar reduction in the growth of *E. gracilis* was observed in both conditions when solar radiation and temperature were low (Fig. 2).

### Chlorophyll (Chl) fluorescence and photosynthetic efficiency

**Figure 4:**
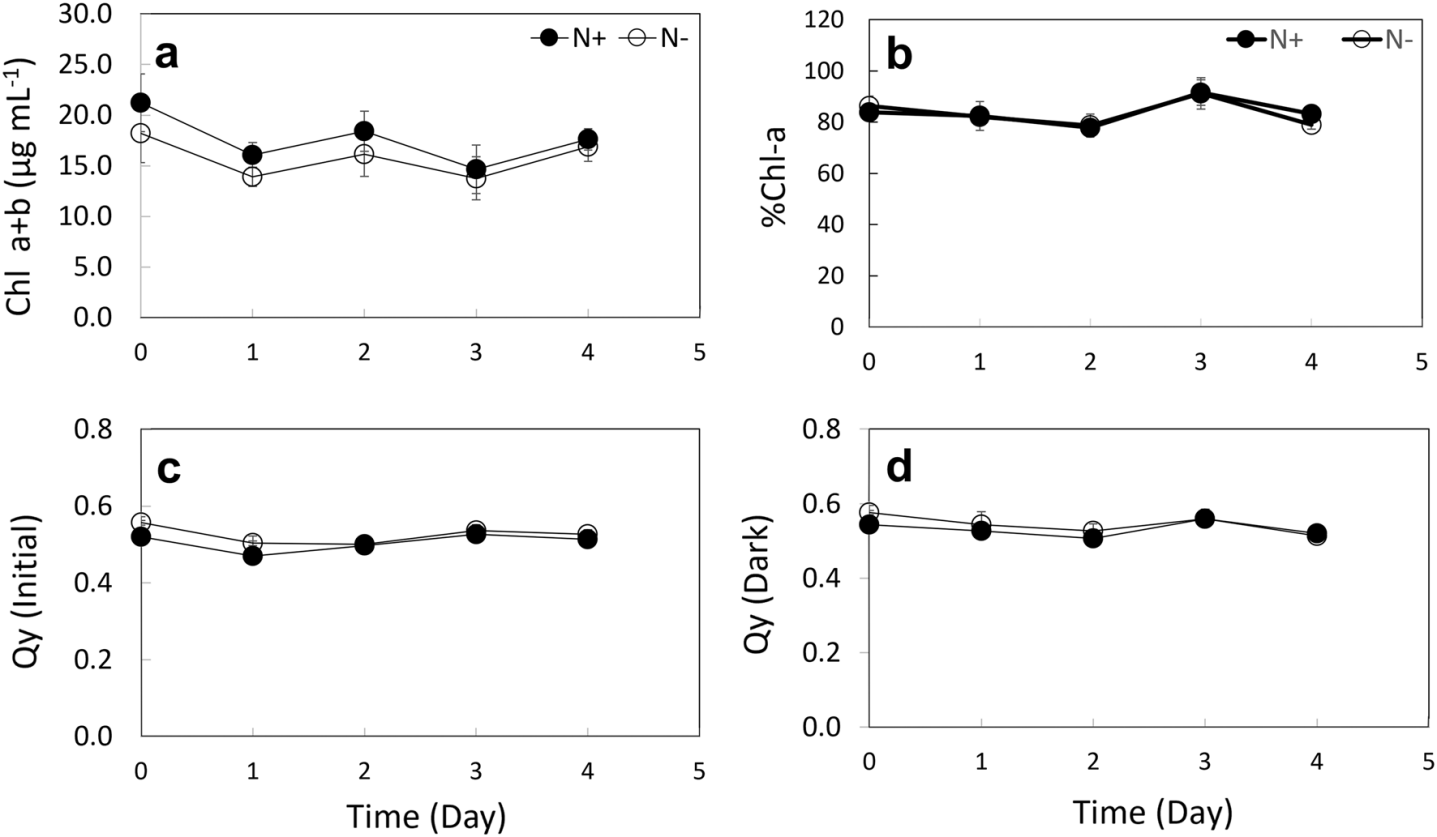
Chlorophyll and photosynthetic activity of *E. gracilis* grown under N-sufficient (N +) and N-starved (N−) conditions. (**a**) Total chlorophyll (Chl) content (Chl a + b) measured during the last week (Week 16) of cultivation. (**b**) Percentage (%) of chlorophyll-a (Chl-a) present in total chlorophyll content. Photosynthetic quantum yield (Qy) measured before (**c**), and (**d**) after 1 h dark incubation. Blank circles represent the N− condition, and filled circles represent the N+ condition. Error bars indicate the standard deviation of the mean values for each treatment (N+ and N−) across three ponds (n = 3).

The study evaluated chlorophyll (Chl) fluorescence and photosynthetic efficiency in response to nitrogen availability. The results showed that the total chlorophyll content (Chl a + b) was slightly lower under N− conditions compared to N+ conditions (Figs. 1b, 4a). However, the chlorophyll-a (Chl-a) composition and Qy response pattern before and after dark incubation were similar in both conditions (Fig. 4b–d). Furthermore, ANOVA analysis for all four measurements indicated no significant difference (*p* > 0.05) between the two conditions.

### Paramylon accumulation, and particulate matter composition

**Figure 5:**
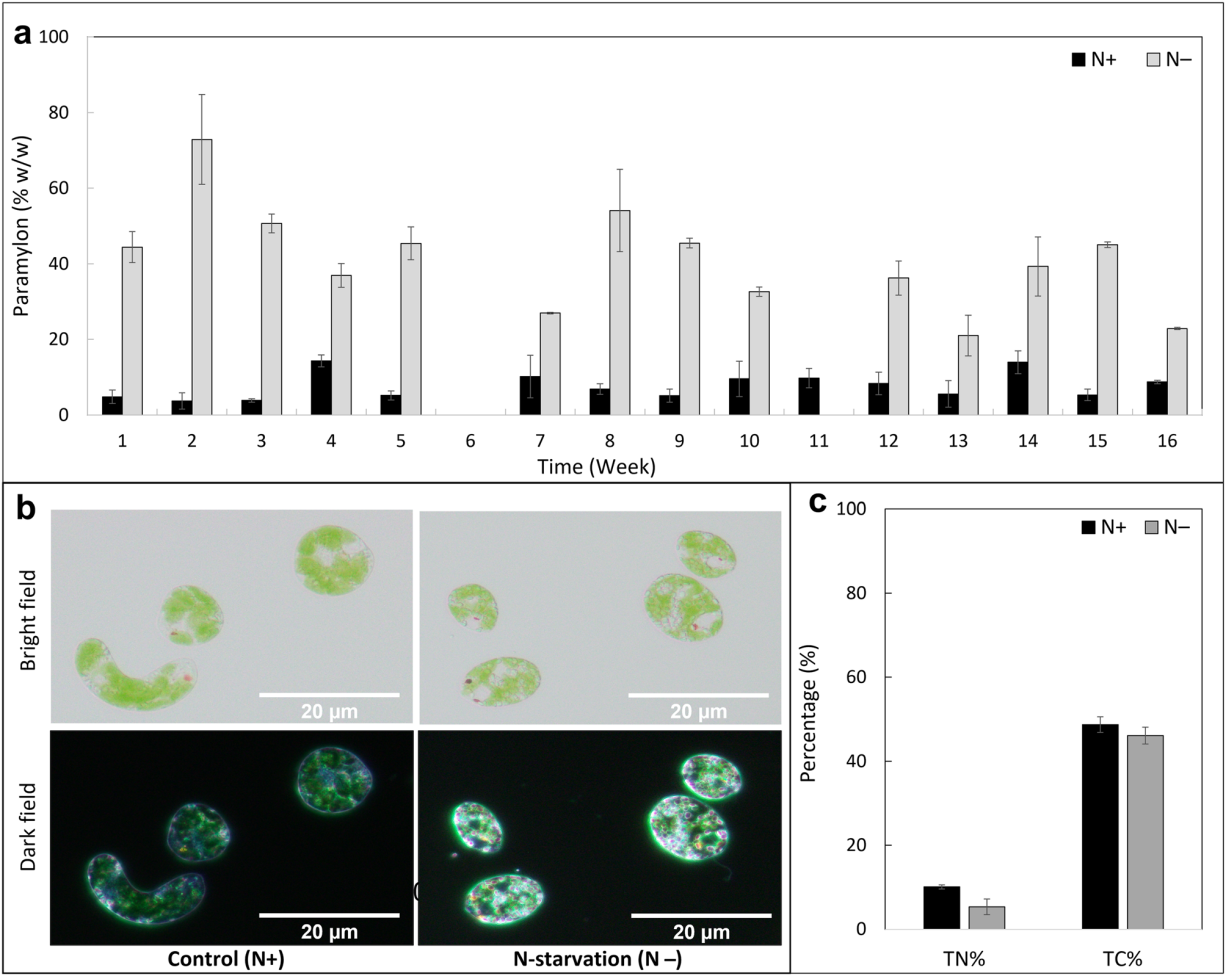
Paramylon and particulate elemental composition. (**a**) Percentage of total carbohydrate content represented as % paramylon (w/w % of dry biomass) in *E. gracilis* cultivated under N-sufficient (N +) and N-starved (N−) conditions. Paramylon content was measured on the last day of each week of the semi-continuous cultivation experiment, except for week 6. Error bars indicate the standard deviation of the mean values of paramylon content for each treatment (N+ and N−) across three ponds (n = 3). (**b**) Microscopy of *E. gracilis* grown in N+ and N− conditions under bright and dark fields. Paramylon bodies can be seen as small transparent or red oval bodies in bright and dark fields, respectively. (**c**) Percentage of total organic carbon (TOC) and nitrogen (TN) measured as an average of three weekly samples taken from one pond for each treatment (N+ and N−) on the last day of each of the three weeks. Error bars indicate the standard deviation of the mean values of TOC and TN measurements for each treatment across the three-week period (n = 3). Grey bars indicate the N− condition, and black bars indicate the N+ condition.

Results showed that the paramylon content was higher in the N− condition compared to the N+ condition (Fig. 5a). Paramylon accumulation ranged from 21.0 to 55.0% of the dry cell weight in N− condition, whereas in N+ condition it was observed to be between 5.0 to 10.0%. On an average 41.0 ± 13.5% (328.8 ± 182.9 pg cell^−1^) of paramylon was accumulated under N− conditions, while only 7.7 ± 3.0% (49.7 ± 23.3 pg cell^−1^) was accumulated under N+ conditions. Paramylon accumulation was also observed under microscopy as small granular bodies in the cells (Fig. 5b).

Total nitrogen (TN) and total carbon (TC) contents were also found to differ between N− and N+ conditions (Fig. 5c). On day-5, higher nitrogen content was observed in the cells under the N+ condition (10.0 ± 0.5%) compared to the N− condition (5.3 ± 1.2%). In contrast, the average N content in the cell-free medium on day-5 was below the detection level around 0 mg L^−1^ in N− condition, and 44.8 ± 13.6 mg L^−1^ in the N+ condition, indicating a higher ratio of nitrogen in the cell to medium in N− compared with N+ condition.

By the end of the weekly experiment, it was found that nearly 48.0 ± 2.0% of carbon was present in the N+ cells, while 46.0 ± 2.0% was present in the N− cells. The total carbon content per cell was 0.4 ± 0.1 ng cell^−1^ in the N− condition and 0.3 ± 0.0 ng cell^−1^ in the N+ condition. Overall, the results suggest that nitrogen availability affects the accumulation of paramylon and the composition of particulate matter in cells.

## Discussion

In this study, we observed a 23% higher growth rate in the N− condition compared to the N+ condition, which cannot be solely explained by the differences in carbon fixation. The cellular responses varied with time. It was clear from the results that during the first three days of the experiment, the cells in both conditions were growing actively under N-sufficient conditions, with similar initial biomass concentrations in all tanks. Therefore, no significant differences were observed in the G-DF1 and G-DF2 tests. However, the growth rate for all days (G-DF3) showed a marked contrast between N+ and N− conditions with a *p*-value less than 0.05, indicating a higher growth rate in N− conditions. The average cell size in N− condition was also smaller than in the N+ condition which was reflected in the D-DF2 and D-DF3 tests with *p*-value less than 0.05. The trend in cell size is consistent with previous reports in other algal genera *Scenedesmus* and *Rhodomonas,* as well as species like *Ankistrodesmus falcatus*, and *Stephanodiscus minululus*^19,35^. Interestingly, correlation analysis showed that the reduction in cell size occurred in both N+ and N− conditions during short cultivation periods, but it appeared to be more pronounced in N− conditions. Additionally, in extended cultivation periods, cell size and number may be impacted by environmental factors beyond nitrogen availability, such as temperature. In the current study low environmental temperatures were found to decrease growth rate and increase cell volume under both conditions. Our results are consistent with previous studies that indicate *E. gracilis* preference for protoplasmic growth at low temperatures and cell division at high temperatures^36^. However, the impact of temperature on cell size in N− conditions appear to be minimal and has not been previously reported. *E. gracilis* in N− condition initially showed a lower cell number and similar diameter compared to N+ condition, while after a few weeks a higher cell number with a smaller diameter was observed, suggesting that prolonged culture in semi-continuous N− conditions modulate the physiology of *E. gracilis*. In N− conditions, *E. gracilis* had to adapt to the environment in which nitrogen availability varies dramatically in a short time. The cells rapidly grow initially due to a sufficient supply of N for 4 days before 3-day N-starvation, but later underwent biochemical changes to store energy^29^. It appears that *E. gracilis* is capable of acclimating to the cyclic process of N-acquisition in N− conditions, resulting in a higher growth rate compared to the N+ condition. Since the cultivation was conducted in an open pond, the environmental conditions in both N+ and N− cultures were similar, the observed differences in growth and biomass were largely due to nitrogen availability.

Under N− conditions, a smaller cell size may suggest a lower protein content^19^, and the carbon pool is directed towards synthesizing carbohydrates or lipids^23,24,25^. In our study, we observed a high (> 40%) paramylon content under N− conditions, indicating that carbon is being fixed into paramylon for energy conservation, which results in an increase in cell density. On the other hand, *E. gracilis* under N+ conditions utilize the fixed carbon to generate energy for cell division, resulting in high cell numbers but low cell density. This is evident from the differences in cell number and biomass between the two conditions during the initial weeks. However, over time, cell numbers were similar under both conditions, while biomass remained higher under the N− conditions. This further supports the idea of *E. gracilis* acclimation, allowing it to grow while accumulating paramylon by utilizing the fixed carbon.

In addition, current results show that the carbon content per cell was almost 1.5 times higher under N− conditions (0.42 ng cell^−1^) than under N+ conditions (0.27 ng cell^−1^). The observed increase in carbon content per cell could also be likely due to an increased carbon fixation through photosynthesis. It is interesting to note that there were no significant differences in the total chlorophyll content despite N-starvation. But from the graph and color of the N− cultures suggest a slight reduction in chlorophyll content. Usually, chlorophyll content decreases under these conditions due to preferential loss of some chloroplast proteins and eventually balancing the photosynthetic process^37^. Moreover, there was no significant difference in the quantum yield before and after dark incubation, therefore it can be inferred that both N+ and N− cultures are equally efficient at converting light energy into chemical energy during photosynthesis. However, the results indicate that the N− cultures can support a higher growth rate and paramylon production, suggesting that the photosynthetic apparatus of these cultures was more efficient and adapted to cope with N starvation. It has been hypothesized previously that microalgae under N-limitation conditions, with lower pigmentation, have high photosynthetic efficiency^38^. This was also observed in sunflower plants where the maximum quantum yield was not influenced under N-deficiency^39^. Moreover, multiple C-fixation pathways (C3, C4, or CAM) pathways have been proven to coexist in some marine algae^40,41^. Among which, the CAM pathway plays a crucial role in maintaining the photosynthesis of plants under stress conditions and can fix carbon during the night. CAM pathway-related metabolites were previously reported to be stimulated by chemical stresses in *E. gracilis*, indicating its existence. Therefore, it is possible that the activation of the CAM pathway under prolonged semi-continuous N-starvation could contribute to active photosynthetic activity and increased C fixation in *E. gracilis*.

Similar results in growth and product yield were observed in a recent study on a *Scenedesmus obliquus* mutant (SO120G) under N− conditions^33^. While chlorophyll content in the current study was not higher than the control, unlike the case with SO120G^33^. Such responses in SO120G were revealed to be a result of the upregulation of genes related to cytochrome b6/f complex (Pet B), and photosynthetic electron transport carrier (Pet J). These proteins mediate the electron transfer from photosystem II to photosystem-I and cyclic electron transport, improving photosynthetic efficiency. In *E. gracilis, pet*B was identified as a part of the operon complex petB-atpB-atpE in the chloroplast genome like land plants^42^. Activation of this gene also induces the expression of the ATP-synthase complex, which is essential for ATP synthesis for the cell and product formation. It is also possible that the slight reduction in Chl content may have improved the light penetration efficiency into the culture and in turn product formation. During the initial hours of nutrient starvation, the rate of product formation will be rapidly induced followed by a gradual decrease^43^. This excess light could supply the energy required for product formation under N-deprived conditions^44^.

On the contrary to high growth in N− condition, a lower growth rate under the N+ condition, is also an important result for understanding biomass productivity. Many microalgal species prefer nitrogen in the form of ammonium, and high concentrations of ammonium in the growth medium enhance algae to absorb NH_4_^+^ ions. However, this also results in excessive NH_4_ flux that can hinder ATP formation and the regulation of photosynthesis^45^. This can lead to ammonium poisoning, where the conversion rate of ammonium to amino acids will be slower than its influx into the cells, thus reducing the growth rate under N+ conditions^46^. In addition, this is accompanied by a decrease in pH, which reduces the efficiency of CO_2_ fixation by the algae. In this study, the pH was consistently maintained between 2.3 to 2.5 in both N+ and N− conditions, and the cells appeared to exhibit active growth, indicating the absence of ammonium poisoning.

The findings of this study, suggest that this cultivation method can improve the yield of *E. gracilis.* and is an important consideration for the economic viability of algae cultivation. The method also reduces the cost of nutrient inputs. Additionally, cultivation of *E. gracilis* using this technique in combination with industrial nutrient wastes, anaerobic digestates of organic wastes, and exhaust CO_2_ from power plants could promote a circular economy and contribute to a clean and sustainable environment^9,47^. Furthermore, this approach may be applied to other microalgae, providing a sustainable source of biofuels and other bioproducts.

However, open pond cultivation presents several limitations^9^. Maintaining a stable year-round cultivation system is challenging, as it depends on favorable weather conditions such as warm temperatures and sufficient sunlight and is thus restricted to specific geographic regions. In this study, a correlation was observed between growth conditions, such as light intensity, temperature, and nitrogen consumption with time, indicating the effects of seasonal variations. Open pond systems are also vulnerable to contamination by other microorganisms like bacteria and other algae, requiring frequent contamination checks. In this study, contamination was not found to be significant when pH was maintained below 2.5. Moreover, high temperatures in open ponds can cause water loss due to evaporation, leading to decreased productivity. Hence, regular monitoring and maintenance of water levels in the ponds are necessary to ensure optimal productivity. Therefore, despite the potential of open pond cultivation, it is important to consider these limitations when designing an algal cultivation system for commercial production.

## Conclusion

In conclusion, this study has demonstrated that *E. gracilis* has a distinctive adaptation mechanism under prolonged N− conditions that were not previously observed. The findings showed a gradual increase in cell numbers, a smaller cell size compared to control conditions and an unaffected photosynthetic activity. Further omics studies are needed to understand the regulatory mechanisms that contribute to these unique observations. The results of this study have promising implications for the industrial-scale production of *E. gracilis* and paramylon using wild-type strain alone. Semi-continuous low-nutrient open pond cultivation can be adopted, which would reduce operating costs while increasing biomass and product yield.

## Materials and methods

### Organism, culture medium, and seed culture preparation

*E. gracilis* maintained at Euglena Co., Ltd. (Tokyo, Japan) was used for the experiment. All experiments including seed scale-up, were conducted using Cramer Myers medium (CM medium)^1,28^ with ammonium sulfate, ((NH_4_)_2_SO_4_), as the nitrogen (N) source at a concentration of 70 mg L^−1^. Initially, 2 L seed cultures were prepared from the stock culture and then scaled up to 30 L in the laboratory. The temperature, aeration, and light intensity for the seed cultures were maintained at 30 °C, 0.01 vv^−1^ min^−1^ (10% CO_2_) and 800 μmol m^−2^ s^−1^ (continuously illuminated by fluorescent lamps), respectively. This culture was further scaled up in open ponds starting from 1 m^2^ and increasing 50 m^2^, 500 m^2^, and finally to a 1000 m^2^ raceway pond. Each scale-up step took approximately 7 days. Prior to the main experiment, the 1000 m^2^ raceway culture was semi-continuously maintained for 4 months with weekly addition of fresh CM medium.

### Study design for the growth experiment

This study was conducted between July 2022 to October 2022, using six 1 m^2^ tanks: R11, R12, R13, R14, R15, and R16. The algal cultures were grown semi-continuously on a weekly cycle, with a 5-day experimental phase followed by a 2-day maintenance phase to allow for sufficient growth until the next cycle. In three tanks (R11, R14, and R15) the cultures were under control condition with sufficient nitrogen (N +) levels, and the other three in R12, R13, and R16 under nitrogen starvation (N–) conditions (Fig. 1). The N-starvation cultivation process involved subjecting the cultures to N− conditions from day 3 to day 5 every week, while the remaining days were maintained under N+ conditions.

### Experiment preparation

At the start of each weekly experiment *(*day 1), the initial biomass concentration in each 1 m^2^ pond was adjusted to about 50 g m^−2^, followed by the addition of the required CM medium without the N source. The ponds were then filled up to a height of 200 mm from the bottom with tap water (total volume of 170 L). For N+ ponds, (NH_4_)_2_SO_4_ was added as an N source at a concentration of approximately 65 mg L^−1^. For N− ponds, the initial ammonium-N concentration was set between 4–8 mg L^−1^ and maintained above 2 mg L^−1^ during the first 2 days of every cycle. After the first two days, the N in the culture was completed consumed by the cells and reaches below the detection level (0 mg L^−1^). The ponds were then left under N− condition for the next 3 days. On day 5 after sampling, 15–30 mg L^−1^ (NH_4_)_2_SO_4_ was added to the N− ponds to maintain the ammonium-N concentration above 2 mg L^−1^ for two days until the next cycle. It is crucial to monitor and maintain the (NH_4_)_2_SO_4_ levels until the N-starvation step in order to prevent cells from experiencing nitrogen starvation prior to the start of the experiment. Weather forecast (temperature, cloudiness and rainfall) was closely monitored for the following days and required amount of (NH_4_)_2_SO_4_ was added accordingly to achieve N-starvation by day-3 in the N− ponds. Same cycle was repeated in the following week. This cycle was repeated weekly until the end of October 2022. Further, the mixing of the culture was maintained by running the two order made paddle wheels at 75 rpm. Aeration with 10% CO_2_ was supplied to all ponds at a rate of 0.01 vv^−1^ min^−1^ and pH was maintained between 2 and 2.5.

### Sampling and measurement

Daily sampling was done at 17:00 h to measure the biomass (dry cell weight), cell number, size, and NH_4_-N content in all the ponds. On every 2nd and last day of the weekly experiment,1.5 L of culture was harvested at 15:00 h and freeze-dried to make powder samples.

### Microscopy, cell number, and size

Cell physiology and contamination were evaluated daily using an OLYMPUS CX-41 Upright Microscope. Cell number and volume were measured using the Sysmex system Particle counter CDA-1000 (Sysmex corp., Hyogo, Japan).

### Dry mass (DM) and productivity

Glass fiber filters (47 mm diameter, pore size ADVANTEC, Toyo Roshi Kaisha, Ltd., Tokyo, Japan) were dried for 2 h (h) at 100 °C in an oven and weighed after cooling. Ten-millilitre sample was filtered through a filter and then rinsed (OR washed) three times with 10 mL of distilled water (Dw). (All filtration steps were performed with gentle vacuum suction.) The wet filter was dried for 2 h at 100 °C before weighing. The biomass productivity was measured by growth rate and specific growth rate, which were calculated as follows;

1 $$\mathrm{Growth rate }\,(\mathrm{g }{\mathrm{m}}^{-2}{\mathrm{d}}^{-1} )={(DM}_{i+1}-{DM}_{i})/v/({t}_{i}-{t}_{i-1})\times\mathrm{ water level}$$

2 $$\mathrm{Specific growth rate }\,\left(\upmu , {\mathrm{d}}^{-1}\right)={(\mathrm{ln}DM}_{i}-{lnDM}_{i-1})/({t}_{i}-{t}_{i-1})$$

where DM_i_ is the dry weight (OR mass) on day t_i_ and v represents the sample amount (10 mL). The values of triplicate measurements were averaged.

### Paramylon analysis

Paramylon concentration was determined according to Ogawa et al. 2015^17^. Observations were measured using a grating-based absorbance reader SH-1300Lab (Corona Electric Co., Ltd., Ibaraki, Japan).

### Environmental parameters

Daily photon flux was recorded using photon flux logger (Eko-photon sensor ML-020P, EKO, Japan), and the water temperature in the ponds was recorded using a temperature logger (Thermo Recorder TR-52i, T&D Corporation, Japan).

### Chlorophyll (Chl) content and photosynthetic efficiency

The total chlorophyll extraction was done from 1 ml *E. gracilis* culture pellet using 100% methanol [Toyama et al., 2019]. For the absorbance measurement, UVmini-1240 spectrophotometer (Shimadzu Co. Ltd., Kyoto, Japan) was used. The total chlorophyll (Chl a + b, µg mL^−1^) content was calculated as follows^48^;

3 $$\mathrm{Chl }a = 16.5*\mathrm{A}665 - 8.3*\mathrm{A}650$$

4 $$\mathrm{Chl }b = 33.8*\mathrm{A}650 - 12.5*\mathrm{A}665$$

5 $$\mathrm{Chl }a + b = 4*\mathrm{A}665 + 25.5*\mathrm{A}650$$

where A665 and A650 represent the absorbance at the wavelength of 665 nm and 650 nm, respectively.

Further, to understand the influence of semicontinuous nutrient starvation on photosynthetic activity, we measured the quantum yield (Qy or F_v_/F_m_). Photosynthetic efficiency (Qy) was measured using AquaPen E-AP 110-C (Environmental Measurement Japan, CO., LTD., Japan). Two fresh samples of 10 ml each were collected from each pond. One sample was wrapped with aluminium foil and kept in dark for 1 h before Qy measurement, while the other one was measured immediately after sampling. Both, Chl content and photosynthetic efficiency were measured during the last two weeks of the whole experiment.

### Particulate matter and Ammonium-nitrogen [NH4-N] measurement

Total Carbon (TC) and total Nitrogen (TN) contents were analyzed by Sumica Chemical Analysis services Ltd., Japan. Dried *E. gracilis* samples collected on day 5 (last day of N− condition) from three consecutive weekly experiments (Week 1 to 3,) were used for the measurement. The average of this data was considered as the maximum available C and N content available in the cells at the end each cycle. NH4-N in the medium was measured using the cell free medium from 1 ml of cell suspension. The sample was first centrifuged at 10,000 rpm for 1.5 min using a high-speed mini centrifuge (GUSTO® HIGH-SPEED MINI CENTRIFUGE, HEA10050, ILLINOIS, USA), and the obtained supernatant was used to measure the NH4-N content using Digital Pack Test Multi SP: DPM-MTSP (Kyoritsu Chemical- Check Lab. Corporation, Japan).

### Data analysis

Statistical analysis was conducted to assess the difference between N− and N+ conditions using R-studio version 4.2.1 software. The growth rate (G), and Diameter (D) data were divided into 3 different groups termed as data frames (DF): DF1, DF2, and DF3. DF1 represents all week’s data from only day 1, DF2 represents data from days 3–5, and DF3 represents all 5-day data. One-way ANOVA was performed on each of the G-DF and D-DF, as well as on chlorophyll content, and Qy. The significant level for all tests was set at 0.05. For growth rate, day 1 was calculated as the difference between day 2 and day 1 according to the equation in (1), and similarly for days 3–5. For cell diameter, the days represent the actual timeline. Additionally, Pearson correlation analysis to examine the correlations between solar radiation, temperature, and time (in days and weeks) with variables such as N content in cells, N+ cell diameter, and N− cell diameter was also performed.

## Supplementary Information

Supplementary Figures.

## Data Availability

All data is provided in the manuscript as figures, tables, and supplemental data.
