# Supplementary material for: New insights into raceway cultivation of Euglena gracilis under long-term semi-continuous nitrogen starvation
Source: Sci Rep. 2023 May 2;13:7123. doi: 10.1038/s41598-023-34164-1 (PMC10154353; doi:10.1038/s41598-023-34164-1)
Supplement: Supplementary file 1 — Supplementary Figures. [file 41598_2023_34164_MOESM1_ESM.pdf]

New insights into raceway cultivation of *Euglena gracilis* under long term semi-continuous nitrogen starvation [20 words]

Ranjith Kumar Bakku[1, 2], Yoshimasa Yamamoto[1, 2], Yu Inaba[1, 2], Taro Hiranuma[1, 2], Enrico Gianino[1, 2], Lawi Amarianto[1, 2], Waleed Mahrous[1, 2], Hideyuki Suzuki*[1, 2], Kengo Suzuki[2, 3]

1. Algae Energy Technology Research Institute, 649-17 Nishiyama, Taki-cho, Taki-gun, Mie 519-2171, Japan
2. Euglena Co., Ltd. G-BASE Tamachi 2nd and 3rd floor, 5-29-11, Shiba, Minato-ku, Tokyo 108-0014, Japan
3. Microalgae Production Control Technology Laboratory, RIKEN 1-7-22, Suehiro, Tsurumi, Yokohama, Kanagawa 230-0045, Japan

Supplementary data

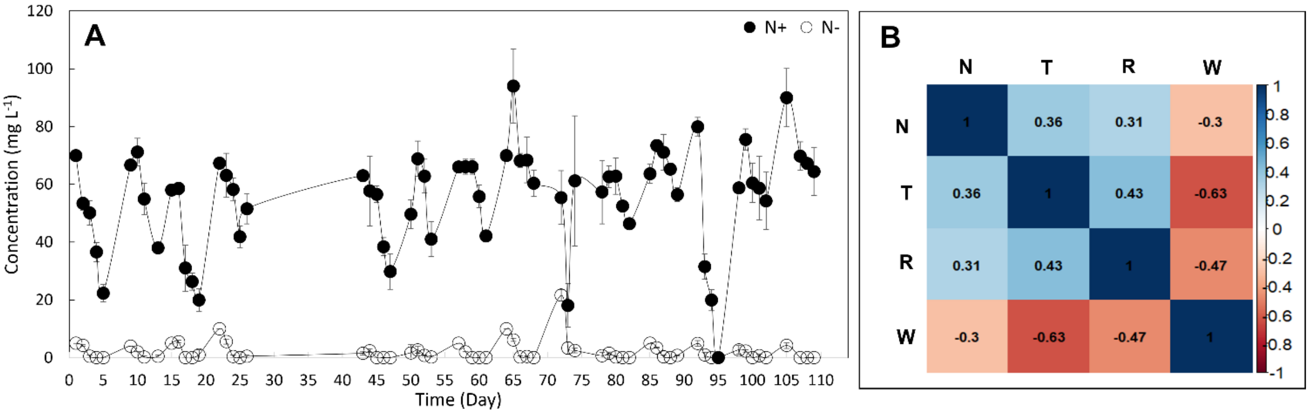

Supplementary Figure 1: Relationship between nitrogen consumption and environmental factors. (**A**) Time-dependent nitrogen concentration in the medium of N+ (N-sufficient) and N– (N-starved) ponds. Empty circles indicate the N– condition, and filled circles indicate the N+ condition. Error bars indicate the standard deviation of the mean nitrogen concentration for each treatment (N+ and N–) across three ponds (n=3). (**B**) Correlation analysis of nitrogen (N) consumption in N+ ponds with temperature, solar radiation (R), and time as a week (W). The color scale represents the strength of the correlation, with blue indicating a positive correlation (1) and red indicating a negative correlation (-1).

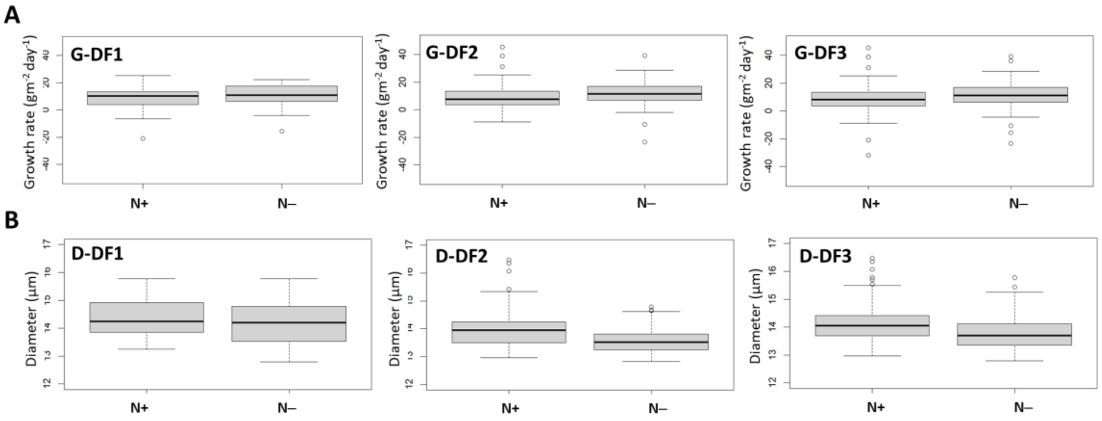

Supplementary Figure 2: Boxplots showing data distribution of growth rate and diameter under N-sufficient (N+) and N-starved (N–) conditions. (A) Data distribution of *E. gracilis* growth rate (G) in data frames G-DF1, G-DF2, and G-DF3, representing data on day 1, days 2-4, and all 5 days, respectively, from 16 weeks of experiments. (B) Data distribution of *E. gracilis* diameter (D) in data frames D-DF1, D-DF2, and D-DF3, corresponding to the same time periods and experiments as the growth rate data frames. "DF" stands for "data frame".

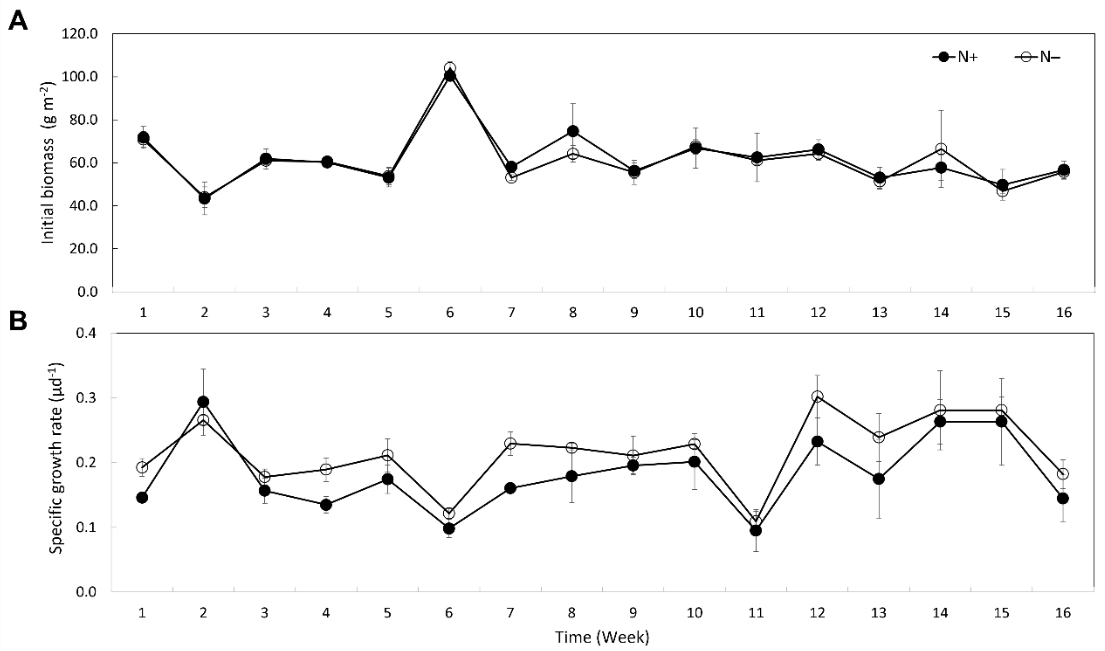

Supplementary Figure 3: Initial biomass concentration, and specific growth rate of *E. gracilis* in N-sufficient (N+) and N-starved (N–) conditions. (A) Weekly average initial biomass concentration in the raceway ponds under N+ and N– conditions. (B) Specific growth rate under N+ and N– conditions. Empty circles indicate the N– condition and filled circles indicate the N+ condition. Error bars indicate the standard deviation of the mean initial biomass or specific growth rate for each treatment (N+ and N–) across three ponds (n=3).

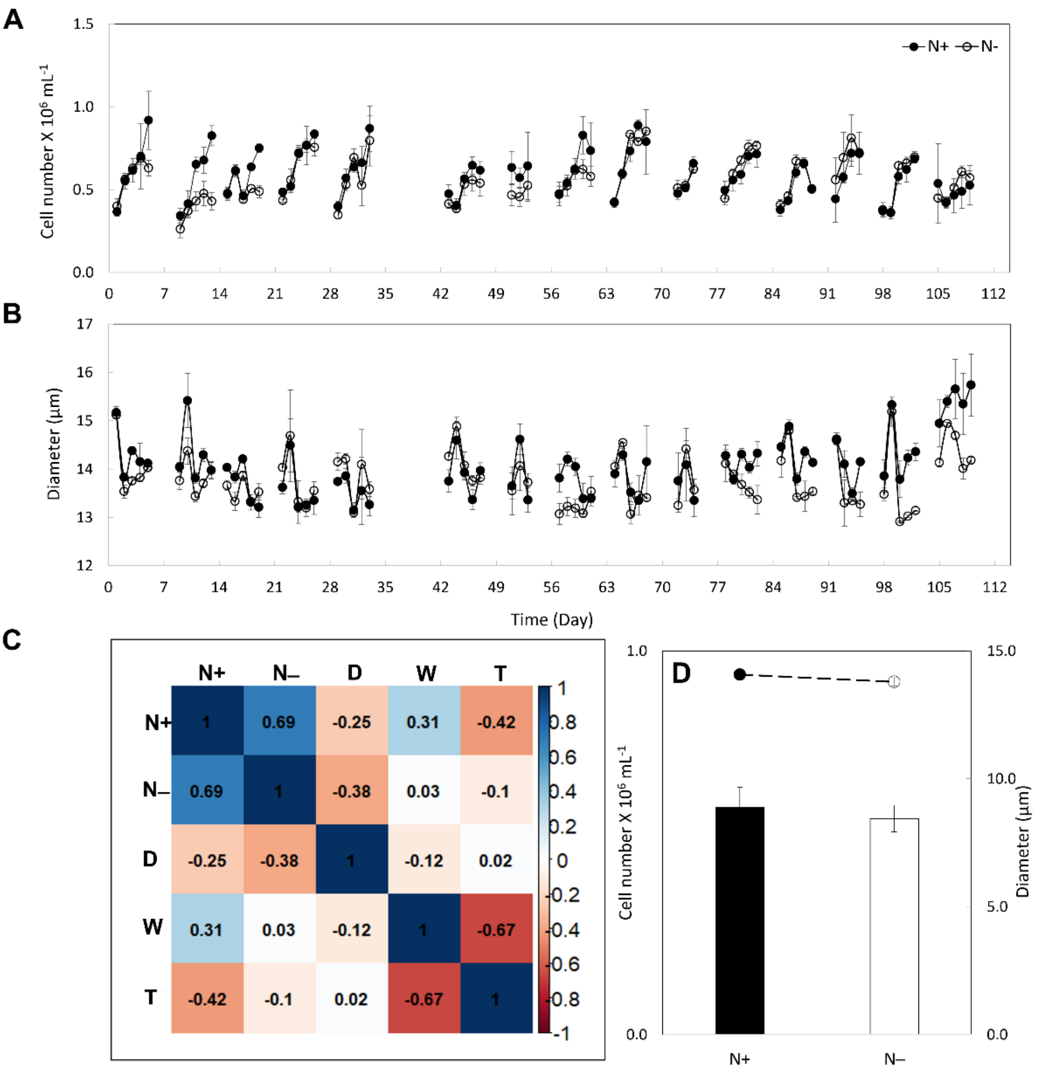

Supplementary Figure 4: Dynamics of *E. gracilis* growth and morphology under (N-sufficient, N+) and N-starved (N–) conditions. (**A**) Daily cell number under N+ and N- conditions. (**B**) Daily cell diameter under N+ and N– conditions. (**C**) Correlation plot showing the relationship between the cell diameter under N+, and N- conditions, day (D), week (W), and temperature (T). Pearson correlation coefficient values are shown. The color scale in D represents the strength of the correlation, with blue indicating a positive correlation (1) and red indicating a negative correlation (-1). (**D**) Average cell number and diameter. Empty circles or bars indicate the N– condition and filled circles or bars indicate the N+ condition. Error bars in A, B, and C indicate the standard deviation of the mean of respective data for each treatment (N+ and N–) across three ponds (n=3).
